# Supplementary material for: Single-cell multiomic human brain atlas reveals regulatory drivers of cortical regionality
Source: Nat Commun. 2026 Feb 21;17:3051. doi: 10.1038/s41467-026-69368-2 (PMC13039890; doi:10.1038/s41467-026-69368-2)
Supplement: Supplementary file 2 — Description of Additional Supplementary Files [file 41467_2026_69368_MOESM2_ESM.pdf]

Description of Additional Supplementary Files

File name: Supplementary Data 1

Description: Donor and sample metadata, subclass information, and subclass marker information by region

File name: Supplementary Data 2

Description: List of region-specific differentially expressed genes across each cell subtype identified by edgeR and overlapping region-specific DEGs with developing brain atlases. Differential expression analysis using edgeR. Differentially expressed genes were identified using two-sided quasi-likelihood F-tests within a negative binomial generalized linear model framework, with P values adjusted for multiple testing using the Benjamini–Hochberg FDR.

File name: Supplementary Data 3

Description: List of region-specific differentially expressed genes across each cell subtype identified by MAST. The default two-sided likelihood ratio test was used, with P values adjusted for multiple testing using the Benjamini–Hochberg FDR.

File name: Supplementary Data 4

Description: List of region-specific cCREs across each cell subtype. Differential cCRE analysis using edgeR. cCREs were identified using two-sided quasi-likelihood F-tests within a negative binomial generalized linear model framework, with P values adjusted for multiple testing using the Benjamini–Hochberg FDR.

File name: Supplementary Data 5

Description: Panel genes and target sequences for DART-FISH profiling

File name: Supplementary Data 6

Description: Correlation of genes across the RC axis by cell subclass using Spearman correlation, which is two-sided, with P values adjusted for multiple comparisons using the Benjamini–Hochberg FDR.

File name: Supplementary Data 7

Description: SCENIC+ eRegulon analysis for all cells with subclass specificity scores for each transcription factor.

File name: Supplementary Data 8

Description: SCENIC+ eRegulon analysis for select neuronal subclasses

File name: Supplementary Data 9

Description: RC activity correlation of identified transcription factors using Spearman correlation, which is two-sided, with P values adjusted for multiple comparisons using the Benjamini–Hochberg FDR.

File name: Supplementary Data 10

Description: Correlation of genes across the TS axis by cell subclass using Spearman correlation, which is two-sided, with P values adjusted for multiple comparisons using the Benjamini–Hochberg FDR.

File name: Supplementary Data 11

Description: TS activity correlation of identified transcription factors using Spearman correlation, which is two-sided, with P values adjusted for multiple comparisons using the Benjamini–Hochberg FDR.
