# Supplementary material for: Single-cell multiomic human brain atlas reveals regulatory drivers of cortical regionality
Source: Nat Commun. 2026 Feb 21;17:3051. doi: 10.1038/s41467-026-69368-2 (PMC13039890; doi:10.1038/s41467-026-69368-2)
Supplement: Supplementary file 14 — Reporting Summary [file 41467_2026_69368_MOESM14_ESM.pdf]

nature portfolio

Corresponding author(s): Kun Zhang, Bing Ren, Jerold Chun, Nathan Zemke

Last updated by author(s): Jan 9, 2026

Reporting Summary

Nature Portfolio wishes to improve the reproducibility of the work that we publish. This form provides structure for consistency and transparency in reporting. For further information on Nature Portfolio policies, see our Editorial Policies and the Editorial Policy Checklist.

Statistics

For all statistical analyses, confirm that the following items are present in the figure legend, table legend, main text, or Methods section.

n/a	Confirmed	
☐	☒	The exact sample size (*n*) for each experimental group/condition, given as a discrete number and unit of measurement
☐	☒	A statement on whether measurements were taken from distinct samples or whether the same sample was measured repeatedly
☐	☒	The statistical test(s) used AND whether they are one- or two-sided<br>*Only common tests should be described solely by name; describe more complex techniques in the Methods section.*
☐	☒	A description of all covariates tested
☐	☒	A description of any assumptions or corrections, such as tests of normality and adjustment for multiple comparisons
☐	☒	A full description of the statistical parameters including central tendency (e.g. means) or other basic estimates (e.g. regression coefficient) AND variation (e.g. standard deviation) or associated estimates of uncertainty (e.g. confidence intervals)
☐	☒	For null hypothesis testing, the test statistic (e.g. *F*, *t*, *r*) with confidence intervals, effect sizes, degrees of freedom and *P* value noted<br>*Give P values as exact values whenever suitable.*
☒	☐	For Bayesian analysis, information on the choice of priors and Markov chain Monte Carlo settings
☒	☐	For hierarchical and complex designs, identification of the appropriate level for tests and full reporting of outcomes
☐	☒	Estimates of effect sizes (e.g. Cohen's *d*, Pearson's *r*), indicating how they were calculated

Our web collection on statistics for biologists contains articles on many of the points above.

Software and code

Policy information about availability of computer code

Data collection	Sequencing data was acquired using an Illumina NovaSeq instrument and associated commercial software. DART-FISH images were acquired using the Leica TCS SP8 confocal microscope with commercial software.
Data analysis	All analysis can be found at the following Github https://github.com/ypauling/human_brain_atlas_cortex_regionality. Tools used are listed and detailed at this resource.

For manuscripts utilizing custom algorithms or software that are central to the research but not yet described in published literature, software must be made available to editors and reviewers. We strongly encourage code deposition in a community repository (e.g. GitHub). See the Nature Portfolio guidelines for submitting code & software for further information.

Data

Policy information about availability of data

All manuscripts must include a data availability statement. This statement should provide the following information, where applicable:

- Accession codes, unique identifiers, or web links for publicly available datasets
- A description of any restrictions on data availability
- For clinical datasets or third party data, please ensure that the statement adheres to our policy

Data generated via SNARE-Seq2 can be found on the NEMO archive via the following link: https://data.nemoarchive.org/biccn/lab/zhang_kun/multimodal/sncell/
Associated metadata can be found in data S1.
Data generated via DART-FISH is currently being uploaded to the BIL archive and will be freely accessible upon publication at the following DOI https://

doi.org/10.35077/g.1179.

Research involving human participants, their data, or biological material

Policy information about studies with human participants or human data. See also policy information about sex, gender (identity/presentation), and sexual orientation and race, ethnicity and racism.

Reporting on sex and gender	Biological sex of each donor has been documented and is reported in the manuscript. Information regarding gender identity was not available. Due to the limited number of available brains covering multiple cortical regions, only one female donor was included, precluding meaningful comparisons between male and female brains.
Reporting on race, ethnicity, or other socially relevant groupings	Information regarding race, ethnicity, or other socially relevant groupings was not provided for the donors. Analyses focused on patterns generalizable across all brains, rather than comparisons between these categories.
Population characteristics	Donor age, sex, and additional relevant information are provided in Supplementary Table 1. Due to the limited number of available tissues, it was not possible to assess other population characteristics, such as race or treatment history.
Recruitment	No patients were actively recruited for this study. It is acknowledged that individuals and families who choose to donate brains to research may represent a distinct subset of the population, which could influence generalizability.
Ethics oversight	All tissue used in this study was obtained with informed consent from donors or their next of kin, in accordance with institutional and national ethical guidelines.

Note that full information on the approval of the study protocol must also be provided in the manuscript.

Field-specific reporting

Please select the one below that is the best fit for your research. If you are not sure, read the appropriate sections before making your selection.

[x] Life sciences [ ] Behavioural & social sciences [ ] Ecological, evolutionary & environmental sciences

For a reference copy of the document with all sections, see nature.com/documents/nr-reporting-summary-flat.pdf

Life sciences study design

All studies must disclose on these points even when the disclosure is negative.

Sample size	No formal sample size calculations were performed. Instead, the study profiled all available tissues, maximizing the number of donors and brain regions to capture biological variability.
Data exclusions	No data were excluded from the analyses. All generated sequencing reads and nuclei passing quality control carefully detailed in the methods section were included in downstream analyses.
Replication	Due to the high cost and resource-intensive nature of multiomic single-nucleus sequencing, formal biological replication was not performed. To capture variability and ensure robustness, the study profiled multiple donors and brain regions. Several regions were sequenced multiple times, and the resulting outputs were pooled as described in the Supplementary Data.
Randomization	During tissue processing, nuclei isolation, and multiomic library preparation, samples were randomized with respect to both brain region and donor to minimize potential batch effects.
Blinding	As this study was based entirely on high-throughput sequencing data, experimental outcomes were not subject to investigator interpretation or manual scoring. Therefore, blinding was not applicable.

Reporting for specific materials, systems and methods

We require information from authors about some types of materials, experimental systems and methods used in many studies. Here, indicate whether each material, system or method listed is relevant to your study. If you are not sure if a list item applies to your research, read the appropriate section before selecting a response.

Materials & experimental systems

n/a	Involved in the study
☒	☐ Antibodies
☒	☐ Eukaryotic cell lines
☒	☐ Palaeontology and archaeology
☒	☐ Animals and other organisms
☒	☐ Clinical data
☒	☐ Dual use research of concern
☒	☐ Plants

Methods

n/a	Involved in the study
☒	☐ ChIP-seq
☒	☐ Flow cytometry
☒	☐ MRI-based neuroimaging

Plants

Seed stocks	*Report on the source of all seed stocks or other plant material used. If applicable, state the seed stock centre and catalogue number. If plant specimens were collected from the field, describe the collection location, date and sampling procedures.*
Novel plant genotypes	*Describe the methods by which all novel plant genotypes were produced. This includes those generated by transgenic approaches, gene editing, chemical/radiation-based mutagenesis and hybridization. For transgenic lines, describe the transformation method, the number of independent lines analyzed and the generation upon which experiments were performed. For gene-edited lines, describe the editor used, the endogenous sequence targeted for editing, the targeting guide RNA sequence (if applicable) and how the editor was applied.*
Authentication	*Describe any authentication procedures for each seed stock used or novel genotype generated. Describe any experiments used to assess the effect of a mutation and, where applicable, how potential secondary effects (e.g. second site T-DNA insertions, mosiacism, off-target gene editing) were examined.*
