# Supplementary figures and images for: Single-cell multiomic human brain atlas reveals regulatory drivers of cortical regionality
Source: Nat Commun. 2026 Feb 21;17:3051. doi: 10.1038/s41467-026-69368-2 (PMC13039890; doi:10.1038/s41467-026-69368-2)

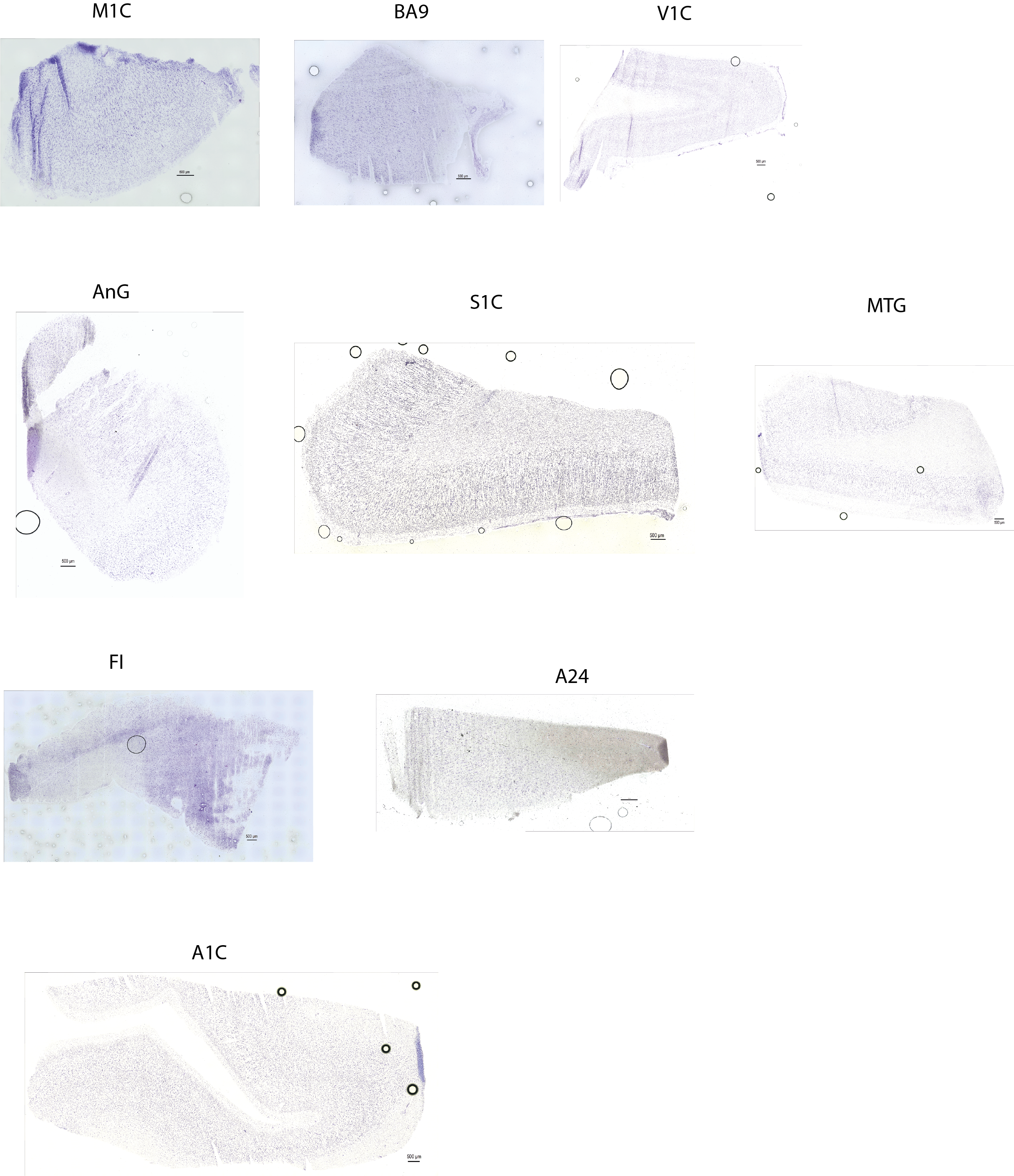

Supplement: Supplementary file 16 — Source data [file 41467_2026_69368_MOESM16_ESM.zip › Source Data Final/Fig1b.png]
